# Tubular Narrowing of Pulmonary Artery Branches by Mediastinal Mass

**Published:** 2014-09-01

**Authors:** Tahereh Davarpasand, Ali Hosseinsabet

**Affiliations:** 1 Cardiology Department, Tehran Heart Center, Tehran University of Medical Sciences, Tehran, IR Iran

**Keywords:** Echocardiography, Pulmonary, Hypertension, Tamponade

## 1. Introduction

**Figure 1.**
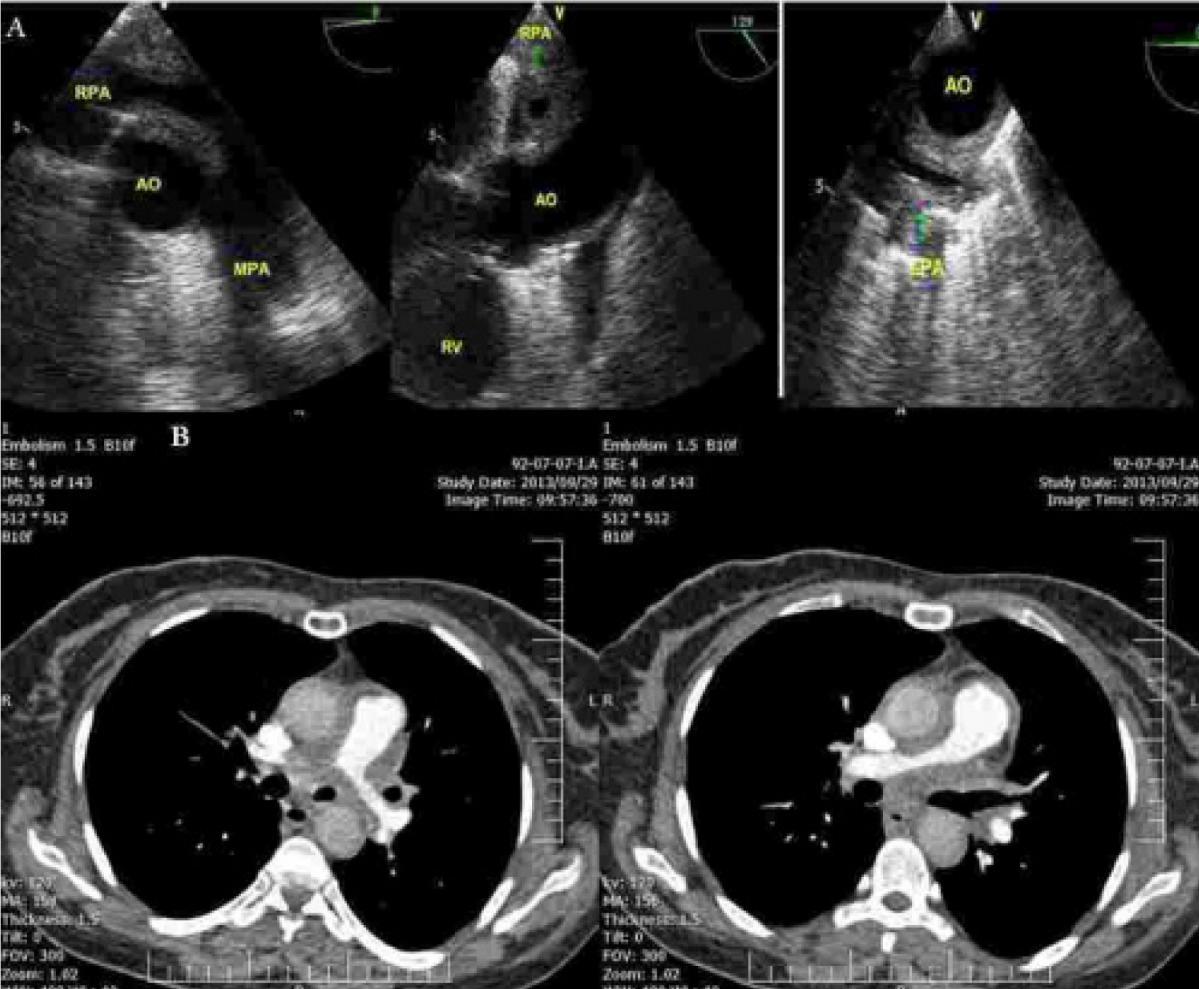
A: Transesophageal Echocardiography Views (Left: Longitudinal View of the Right PA Showing Tubular Narrowing, Middle: Cross Sectional View of the Right PA Showing Concentric Narrowing, Right: Longitudinal View of the Left PA Showing Tubular Narrowing); B: CT Angiography Slices of PA Branches (Left: Tubular Narrowing of LPA by Mediastinal Mass, Right: Tubular Narrowing of RPA by Mediastinal Mass) AO, aorta; RV, right ventricle; MPA, main pulmonary artery; RPA, right pulmonary artery; LPA, left pulmonary artery

A 42-year old woman presented with dyspnea on exertion, exertional chest pain NYHA functional class II, weight loss, and anorexia since 2 months ago. Physical examinations were in favor of tamponade, documented by emergent echocardiography; therefore, open drainage of pericardial fluid was done. After that, transthoracic echocardiography showed moderate right ventricular enlargement with mild systolic dysfunction. Also, there were mild tricuspid regurgitations with 57 mmHg gradient and turbulent flow Pulmonary Artery (PA) bifurcation, extended to its branches. Left ventricle size and function were normal. In transesophageal echocardiography, PA branches were concentrically narrowed from the origin to distal part (6 millimeters in diameter) with turbulent flow probably due to external compression of the surrounding mass (Figure 1A, Video1-6 (To see the videos, refer to the html Version)). PA CT angiography showed no sign of thromboemboli and amorphous low density mediastinal soft tissue surrounding narrow PA branches (Figure 1B). Besides, tuberculin test was negative. *What was the diagnosis in transesophageal echocardiography and PA CT angiography?*

## 2.Answer

Using transesophageal echocardiography and PA CT angiography, the entity of mediastinal mass was not clear perfectly. Therefore, thoracotomy was done and the results showed enlarged lymph nodes. In addition, pathological examinations showed reactive follicular hyperplasia with no granuloma formation.

## 3.Comment

External compression of PA by mediastinal lymph nodes has been reported (1-3), with lymphoma (3) and sarcoidosis (1) being the most important etiologies. However, lymphoma and sarcoidosis were not detected in our case. Therefore, reactive lymphoadenopathy might have been responsible for PA branches stenosis. PA branches stenting can be a treatment option in this case (2).

## Supplementary Material

Supporting Movie
